# Updates in diabetic peripheral neuropathy

**DOI:** 10.12688/f1000research.7898.1

**Published:** 2016-04-25

**Authors:** Kelsey Juster-Switlyk, A. Gordon Smith

**Affiliations:** 1 Department of Neurology, University of Utah School of Medicine, Salt Lake City, UT, USA

**Keywords:** diabetic peripheral neuropathy, Diabetes mellitus, polyneuropathy

## Abstract

Diabetes has become one of the largest global health-care problems of the 21 ^st^ century. According to the Centers for Disease Control and Prevention, the population prevalence of diabetes in the US is approaching 10% and is increasing by 5% each year. Diabetic neuropathy is the most common complication associated with diabetes mellitus. Diabetes causes a broad spectrum of neuropathic complications, including acute and chronic forms affecting each level of the peripheral nerve, from the root to the distal axon. This review will focus on the most common form, distal symmetric diabetic polyneuropathy. There has been an evolution in our understanding of the pathophysiology and the management of diabetic polyneuropathy over the past decade. We highlight these new perspectives and provide updates from the past decade of research.

## Introduction

Diabetes has become one of the largest global health-care problems of the 21 ^st^ century. The number of people with diabetes worldwide is predicted to double between 2000 and 2030, reaching a pandemic level of 366 million people ^1^. Diabetic polyneuropathy (DPN), which has a lifetime prevalence of approximately 50%, is the most common diabetic complication ^2– 6^. DPN is a leading cause for disability due to foot ulceration and amputation, gait disturbance, and fall-related injury. Approximately 20 to 30% of patients with DPN suffer from neuropathic pain ^5– 8^. DPN significantly lowers quality of life and substantially increases health costs associated with diabetes ^9^. The total annual medical costs for diabetes is $6,632 per patient ^10^. Those with DPN experience a twofold increase in health-care costs ($12,492), and those with severe painful peripheral neuropathy experience a fourfold increase ($30,755) ^10^. On a larger scale, the annual cost of diabetes in the US in 2012 was $245 billion, and it has been estimated that about 27% of health-care costs of diabetes can be attributed to DPN ^11, 12^. Despite a long history of research in this area, we are only starting to understand the pathophysiology of the disease. The past decade of research is marked by many surprises, the most pertinent being the major differences in pathogenesis and treatment of DPN in type 1 and type 2 diabetes.

## Clinical features

Diabetes causes a wide variety of acute, chronic, focal, and diffuse neuropathy syndromes. By far the most common is DPN, which accounts for 75% of diabetic neuropathy and thus is the focus of our review ^4, 13^. The other patterns of nerve injury include diabetic autonomic neuropathy, cranial neuropathy, mononeuritis multiplex, mononeuropathy, radiculoplexus neuropathies, diabetic neuropathic cachexia, and treatment-induced neuropathy in diabetes ^14^. The last of these is an important recent advance and will be discussed separately. A patient may have multiple forms of neuropathy.

DPN has been defined by the Toronto Consensus Panel on Diabetic Neuropathy as a “symmetrical, length-dependent sensorimotor polyneuropathy attributable to metabolic and microvessel alterations as a result of chronic hyperglycemia exposure and cardiovascular risk covariates” ^4^. Sensory symptoms start in the toes and over time affect the upper limbs in a distribution classically described as a “stocking and glove” pattern. Motor involvement is not typically seen in the early stages of DPN. Patients describe a range of sensory symptoms, which may include loss of pain sensation or “Novocain-like” insensitivity, tingling, “pins and needles” sensation, burning, “electric shocks”, allodynia (painful sensation to an inoffensive stimuli), or hyperalgesia (increased sensitivity to painful stimuli). Interestingly, symptoms are not a predictable indicator of the severity of axonal loss. Often those with the most severe painful symptoms have minimal or no sensory deficit on exam or electrodiagnostic studies ^4^. Neuropathic pain affects up to 20 to 30% of patients with DPN and is one of the main reasons this group seeks medical care ^5– 8^.

## Treatment-induced neuropathy in diabetes

In a seemingly paradoxical relationship, both poor glucose control and rapid treatment of hyperglycemia can be associated with an increased risk of neuropathy. A clinically distinct form of neuropathy that deserves mention is treatment-induced neuropathy in diabetes (TIND). This underdiagnosed iatrogenic small-fiber neuropathy is defined as the “acute onset of neuropathic pain and/or autonomic dysfunction within 8 weeks of a large improvement in glycemic control specified as a decrease in glycosylated HbA1c of more than 2% points over 3 months” ^15^. TIND was first recognized soon after the introduction of insulin and named “insulin neuritis” ^16^. For many decades, “insulin neuritis” was considered a rare cause for acute neuropathy. However, recently published data suggest that it is much more common and clinically relevant. It is most common in type 1 diabetes mellitus (DM) treated with insulin, although rapid glucose correction can occur in both types of diabetes as a result of either insulin, or less frequently, oral agents. In a study by Gibbons and Freeman, a surprising 10.9% of 954 subjects with diabetes met criteria for TIND, and the risk of developing TIND was associated with the magnitude and rate of HbA1c change ^15^. Similar to DPN, the neuropathy of TIND generally follows a length-dependent pattern, but, in contrast, the pain and autonomic symptoms are more extensive and less responsive to opioids. The underlying pathophysiology is poorly understood, although it has been suggested that rapid glycemic control both with and without insulin leads to hemodynamic changes (arteriovenous shunting) resulting in endoneurial hypoxia of small fibers ^17, 18^.

## Diagnosis

The diagnosis of a DPN is most often made on clinical grounds with a suggestive clinical history and neurologic exam. The Toronto consensus criteria define probable neuropathy as the presence of two or more of the following: neuropathic symptoms, decreased distal sensation, or decreased or absent ankle reflexes. Confirmed neuropathy requires abnormality of nerve conduction study (NCS) or a validated measure of small-fiber function ^19^. However, the diagnostic value of NCS in routine clinical practice has been called into question. Whereas patients with warning signs for an atypical neuropathy (e.g., acute onset, asymmetry, proximal involvement, and unexpected severity) clearly need electrodiagnostic testing, those with typical DPN likely do not need NCS to confirm diagnosis ^20– 22^. Early diabetic neuropathy often preferentially involves small-diameter axons; thus, skin biopsy with assessment of intraepidermal nerve fiber density (IENFD) may be useful in confirming the diagnosis when clinically warranted. In our practice, we use skin biopsies when we suspect a predominately small-fiber neuropathy in patients with an atypical course or a paucity of risk factors. Corneal confocal microscopy provides a non-invasive quantitative method of detecting neuropathy, and has been found to be more sensitive in assessing nerve repair than other standard measures such as IENFD and NCS ^23^. Patients with suspected DPN should have a basic workup, including a blood glucose or hemoglobin A1c to confirm diabetes (fasting plasma glucose of more than 126 mg/dL or A1c of more than 6.5%) or pre-diabetes (fasting plasma glucose of more than 100 mg/dL ****or A1c of 5.7 to 6.4%), vitamin B _12_ deficiency, paraproteinemia (serum protein electrophoresis and immunofixation), and (when appropriate) evaluation for alcohol use ^24, 25^. When routine blood glucose testing is normal, the glucose tolerance test should be considered ^24^. An important cause of vitamin B _12_ deficiency is iatrogenic, linked to cumulative doses of metformin ^26^.

## Pathogenesis of diabetic polyneuropathy

Despite the different pathophysiology underlying type 1 and type 2 diabetes, there has been a longstanding assumption that the mechanism leading to DPN is shared. This assumption has recently been called into question ^8^. Type 2 DM is much more common (90 to 95%) but has a slightly lower lifetime incidence of neuropathy (45%) compared with the 54 to 59% associated with type 1 DM ^27^. Whereas treating hyperglycemia in type 1 DM can significantly reduce the incidence of neuropathy by up to 60 to 70% ^28, 29^, glucose control in type 2 DM has only a marginal 5 to 7% reduction in the development of neuropathy ^30, 31^. Over 40% of patients with diabetes develop neuropathy despite good glucose control, suggesting that other factors are driving nerve injury ^32^. Type 2 DM is inseparably linked to the obesity epidemic; about 90% of diabetic risk is attributable to excess weight ^1^. The longstanding notion that DPN occurs only after longstanding hyperglycemia has been replaced by the observation that even those with good glycemic control (HbA1c of less than 5.4%) are at risk ^33^. Many recent studies have implicated cardiovascular risk factors, including obesity ^34^, hypertriglyceridemia, hypercholesterolemia, hypertension, and cigarette smoking, in the pathogenesis of DPN ^35^.

The pathogenesis of diabetic peripheral neuropathy is complex and is marked by both metabolic and vascular factors ^36^. Hyperglycemia is only one of the many key metabolic events known to cause axonal and microvascular injury. A comprehensive, but by no means exhaustive, list of key players include hyperglycemia, toxic adiposity, oxidative stress, mitochondrial dysfunction, activation of the polyol pathway, accumulation of advanced glycation end products (AGEs), and elevation of inflammatory markers ^2, 35^. Although nerve fiber loss is accepted as the genesis of insensitivity in DPN ^4^, the pathophysiological explanation behind neuropathic pain in diabetes is poorly understood. Sural nerve biopsies from patients with DPN revealed microvascular defects, including endoneurial basement membrane thickening as well as endothelial cell proliferation and hypertrophy, findings which were absent in diabetics without DPN ^37^.

## Management

Management of DPN includes attempts to alter the natural history and symptomatic treatments. The Diabetes Controls and Complications Trial clearly demonstrated that aggressive glycemic control reduced the risk of DPN and rate of progression of DPN in patients with type 1 diabetes ^28^. Although for many years it was assumed that the same was true for DPN associated with type 2 diabetes, multiple studies now show no meaningful impact on DPN risk with aggressive versus standard glycemic control ^38^. Owing to a growing understanding of the association between metabolic syndrome and DPN, more emphasis has been placed on obesity (particularly, visceral adiposity), dyslipidemia, and hypertension ^8^. Several small studies suggest that lifestyle changes, including diet and exercise, may slow the progression of neuropathy by promoting small nerve fiber regeneration in neuropathy patients with diabetes and prediabetes ^39^. One year of exercise has been shown to result in increased IENFD in diabetic patients without neuropathy, suggesting that this approach may be a useful preventative therapy ^39^. This is supported by the observation, based on NCSs, that long-term exercise training can help prevent the development of DPN ^40^. Data from a study using a capsaicin axotomy regeneration experiment before and after exercise suggest that the beneficial effects of exercise are mediated by enhanced nerve regeneration ^41^. A common feature of these studies is individualized counseling or supervised exercise with clear goals and accountability. Integral to management of DPN is prevention of diabetic complications such as ulcers and falls. The lifetime risk of developing foot ulcers in patients with diabetes is high at 15% ^2^. In addition to modifying metabolic factors contributing to the underlying diabetes, effective interventions to prevent ulceration include educating patients about prescription footwear, periodic foot examinations, and intensive podiatric care. Falls are an under-reported cause of injury, emergency room visits, and loss of independence. Primary prevention should start with primary care providers and involve a fall risk assessment, education, and referral to physical therapy or a community exercise program when appropriate ^42^.

Many potential disease-modifying drugs have been designed to target multiple metabolic pathways, including reactive oxygen species inhibitors, aldose reductase inhibitors, protein kinase C-beta inhibitors, agents acting on the AGE pathway, and agents acting on the hexosamine pathway ^2, 43^. Despite very promising preclinical and early-phase clinical data, none of these has proven effective. Given the known importance of oxidative stress in the underlying endothelial dysfunction and microvascular complications in diabetic neuropathy, one would expect antioxidants to be a viable therapeutic option. Alpha lipoic acid (ALA) is considered the most successful antioxidant in clinical trials and has been approved for the treatment of DPN in Europe but not in the US ^6, 44^. In the NATHAN 1 (Neurological Assessment of Thioctic Acid in Diabetic Neuropathy 1) trial, a multicenter randomized double-blind trial, 600 mg ALA daily for 4 years did not influence the primary composite end point (exam findings, a symptoms score, and NCSs) but did show a benefit in the examination score, but not NCSs ^43^. In our practice, we have found some benefit in using ALA 600 mg daily for mild painful neuropathies or as an adjunct in more moderate or severe cases. Aldose reductase treatment showed initial promise in the treatment of DPN in several studies ^45, 46^; however, in a Cochrane review of 32 randomized controlled trials, no statistically significant difference between aldose reductase inhibitors and placebo was found ^47^. Many other disease-modifying therapies have been studied, although the vast majority, including the antioxidant benfotiamine, have failed to show efficacy ^48^.

The most common disabling symptom of DPN is pain, which occurs in 20 to 30% of patients ^5– 8^. The most popular analgesic treatments include tricyclic antidepressants (amitriptyline and nortriptyline), anticonvulsants (gabapentin and pregabalin), and serotonin-norepinephrine reuptake inhibitors (SNRIs) (duloxetine and venlafaxine) ^2^. The European Federation of Neurological Societies (EFNS) and the American Academy of Neurology (AAN) have each published evidence-based guidelines regarding treatment of painful DPN. However, given the relative paucity of head-to-head trials on comparative efficacy and the short duration of most clinical trials, clinicians rely heavily on clinical judgment based on a patient comorbidities, potential adverse effects, medication interactions, and cost ^49, 50^. Despite evidence suggesting that the effectiveness of these first-line agents does not differ substantially, only duloxetine and pregabalin are approved by the US Food and Drug Administration to treat neuropathic pain in diabetes ^5, 50^. These two agents are significantly more expensive than other first-line agents. A recent cost comparison found that the costs for 1 month at the typical starting dose were $189.98 for pregabalin and $170.99 for duloxetine compared with $18.99 for gabapentin and $12.99 for amitriptyline ^50^. The EFNS and AAN provide conflicting evidence for topical agents and in clinical practice they are rarely sufficient as monotherapy. Both controlled-release oxycodone and tramadol with acetaminophen were recommended with level A evidence by the EFNS and with level B evidence by the AAN ^8^. A recent survey showed that DPN was treated with anticonvulsants in 27%, SNRIs in 18%, and opioids in 43% ^51^. Despite many effective agents, poor access to specialists leads to inadequate and often inappropriate treatment.

Beyond pain, falls and foot ulcers are two very costly and debilitating symptoms, which are important patient-oriented outcomes. Patients with DPN are two to three times more likely to fall than diabetics without neuropathy. This is not a late-stage complication; the increased risk of falls has been noted 3 to 5 years prior to their diagnosis ^8, 52^. In addition, those with neuropathy have a 15% increased risk of developing ulcers during their disease course and 6 to 43% of those with ulcers will eventually have an amputation ^8^.

## Conclusions

DPN is a common and costly disease. Over the past decade, there have been great strides in understanding the underlying pathophysiology and the interplay of metabolic risk factors. Aggressive glycemic control is an effective disease-altering strategy in type 1 diabetes but not in type 2 diabetes. The many metabolic and inflammatory consequences of toxic adiposity are likely major contributors to neuropathy risk, particularly in type 2 diabetes. Evolving data suggest that weight loss and exercise are helpful strategies for patients with neuropathy in the setting of both diabetes and prediabetes. Implementing strategies that target these modifiable risk factors will require fundamental social changes and may necessitate major public health initiatives.
